# Structural basis of vitamin C recognition and transport by mammalian SVCT1 transporter

**DOI:** 10.1038/s41467-023-37037-3

**Published:** 2023-03-13

**Authors:** Mingxing Wang, Jin He, Shanshan Li, Qianwen Cai, Kaiming Zhang, Ji She

**Affiliations:** 1 MOE Key Laboratory for Cellular Dynamics, Hefei National Research Center for Interdisciplinary Sciences at the Microscale, School of Life Sciences, Division of Life Sciences and Medicine, University of Science and Technology of China, Hefei, 230026 China; 2 Center for Advanced Interdisciplinary Science and Biomedicine of IHM, Division of Life Sciences and Medicine, University of Science and Technology of China, Hefei, 230026 China; 3 Biomedical Sciences and Health Laboratory of Anhui Province, University of Science and Technology of China, Hefei, 230026 China

**Keywords:** Cryoelectron microscopy, Membrane proteins, Permeation and transport

## Abstract

Vitamin C (L-ascorbic acid) is an essential nutrient for human health, and its deficiency has long been known to cause scurvy. Sodium-dependent vitamin C transporters (SVCTs) are responsible for vitamin C uptake and tissue distribution in mammals. Here, we present cryogenic electron microscopy structures of mouse SVCT1 in both the apo and substrate-bound states. Mouse SVCT1 forms a homodimer with each protomer containing a core domain and a gate domain. The tightly packed extracellular interfaces between the core domain and gate domain stabilize the protein in an inward-open conformation for both the apo and substrate-bound structures. Vitamin C binds at the core domain of each subunit, and two potential sodium ions are identified near the binding site. The coordination of sodium ions by vitamin C explains their coupling transport. SVCTs probably deliver substrate through an elevator mechanism in combination with local structural arrangements. Altogether, our results reveal the molecular mechanism by which SVCTs recognize vitamin C and lay a foundation for further mechanistic studies on SVCT substrate transport.

## Abstract (web summary)

Sodium-dependent vitamin C transporters are responsible for vitamin C uptake and tissue distribution in mammals. Here, authors present cryo-EM structures of mouse SVCT1 in both the apo and substrate-bound states, revealing the structural basis of substrate recognition and transport.

## Introduction

Vitamin C is essential for human health and participates in numerous physiological processes, including collagen hydroxylation^1–3^, hormone synthesis^3,4^, the demethylation of DNA and histones^5^, hypoxia-inducible factor hydroxylase-mediated oxygen sensing^6^, free radical scavenging^7^, and immune regulation^8^. In these processes, vitamin C functions as an enzymatic cofactor or an antioxidant. For example, vitamin C is a cofactor for prolyl and lysyl hydroxylases that hydroxylate collagen^1,2^, and vitamin C deficiency causes collagen instability and scurvy^9^, a disease characterized by muscle weakness, tooth loss and impaired wound healing^10^.

Humans are not able to synthesize ascorbic acid de novo and need to absorb exogenous vitamin C from the diet due to the inactivation mutation of the gene encoding the gulono-gamma-lactone oxidase that catalyzes ascorbic acid synthesis from glucose^11^. Membrane transporters are thus required for the uptake of vitamin C into the body. While glucose transporters (GLUTs), including GLUT1, GLUT3 and GLUT4, take up dehydroascorbic acid (the oxidized form of vitamin C) by facilitating diffusion^12–14^, sodium-dependent vitamin C transporters (SVCTs) mediate the active uptake of L-ascorbic acid (the reduced form) with high substrate specificity^15–21^. Three different isoforms, SVCT1-3, encoded by *SLC23A1-3*, have been identified in the human SVCT family. SVCT1 and SVCT2 are symporters that cotransport sodium and vitamin C with a 2:1 stoichiometry down electrochemical Na^+^ gradients across cell membranes^17,18^. SVCT1 is primarily expressed in epithelial tissues, such as the intestine and kidney, and plays a key role in maintaining the whole-body homeostasis of vitamin C^10,15,22^. In contrast, the widely expressed SVCT2 distributes vitamin C to various tissues^10,15,19^, including the brain, retina, placenta, spleen, and prostate. Both *Slc23a1* and *Slc23a2* knockout mice showed high perinatal mortality^23,24^. SVCT3 does not transport vitamin C, and its function is still unknown^10^.

SVCTs belong to the nucleobase-ascorbate transporter (NAT) family^25,26^, the members of which transport a variety of physiological substrates, including xanthine, uric acid, uracil, and vitamin C. Substrate specificity and transport of NAT proteins were suggested to be related to a signature motif with a conserved sequence [Q/E/P]-N-X-G-X_4_-T-[R/K/G] (X indicates a nonspecific amino acid)^27^ (Supplementary Fig. 1). Previous structural studies of the bacterial uracil transporter UraA and the fungal xanthine transporter UapA provided important insights into nucleobase recognition and transport^28–30^. However, mammalian SVCTs specifically catalyze sodium-dependent vitamin C transport.

In this work, we present the cryogenic electron microscopy (cryo-EM) structures of MmSVCT1 in both the apo and substrate-bound states, revealing the structural basis of vitamin C binding and transport.

## Results

### Overall structure of MmSVCT1

**Fig. 1:**
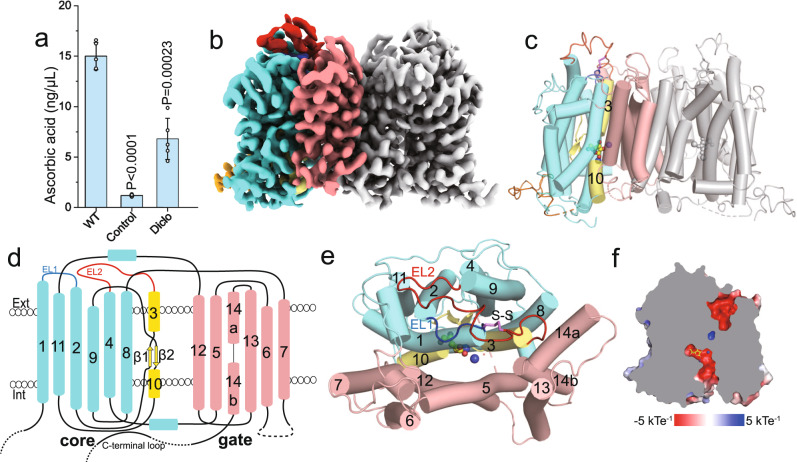
Overall structure of MmSVCT1. **a** Ascorbic acid uptake activities of MmSVCT1. WT, wild-type; Diclo, diclofenamic acid. Data points are the mean ± SEM (*n* = 5 independent experiments). *P* values from the two-tailed *t*-test are indicated on the bar chart. Source data are provided as a Source Data file. **b**, **c** Cryo-EM density map (**b**) and cartoon representation (**c**) of the MmSVCT1 dimer with one subunit colored by individual elements and the other colored gray. Vitamin C is shown as spheres. **d** Domain topology of the MmSVCT1 subunit. **e** One MmSVCT1 subunit viewed from the top colored as in **b**–**d**. The disulfide bond between Cys78 and Cys136 is marked by S‒S. EL1 and EL2 are colored blue and red, respectively. **f** Slab view of the surface electrostatic potential of MmSVCT1 showing the inward-open conformation.

MmSVCT1 was recombinantly expressed in HEK293F cells, and its activity was examined by an in vitro ascorbic acid transport assay (Methods). In this assay, cells were first incubated with ascorbic acid and then washed and lysed to release the imported ascorbic acid, which reduces Fe^3+^ to Fe^2+^, resulting in a colorimetric product with a characteristic absorbance at 593 nm. Cells overexpressing wild-type MmSVCT1 displayed robust ascorbic acid uptake compared to control cells (Fig. 1a). Diclofenamic acid, an SVCT inhibitor^31^, efficiently inhibited transport activity (Fig. 1a), verifying that the uptake of ascorbic acid was mediated by MmSVCT1.

MmSVCT1 structures were determined in the presence and absence of L-ascorbic acid to overall resolutions of 3.2 and 3.5 Å, respectively, using single-particle cryo-EM (Fig. 1b, c, Supplementary Figs. 2–4 and Supplementary Table 1). The structures in the apo and substrate-bound states were highly similar with an RMSD of 0.53 over 894 Cα atoms (Supplementary Fig. 5), so the 3.2-Å vitamin C-bound structure is referred to for most of the description. Similar to other NAT transporters^29,30^, MmSVCT1 forms a homodimer (Fig. 1b, c), in which each MmSVCT1 subunit consists of 14 transmembrane segments (TM1–TM14) (Fig. 1d, e). TM1–TM7 and TM8–TM14 are inverted structural repeats related by a pseudo twofold axis parallel to the membrane (Supplementary Fig. 6). The transmembrane domain is spatially separated into a gate domain (TM5–TM7 and TM12–TM14) and a core domain (TM1–TM4 and TM8–TM11) (Fig. 1d, e). Transmembrane helices from the gate domain, including TM5, TM6, and TM12 from one subunit and TM13 from the neighboring subunit, form the dimeric interface of MmSVCT1 largely through hydrophobic interactions and van der Waals contacts (Supplementary Fig. 7). Lipid binding was previously shown to be important for UapA dimer formation^32^, but the lipid binding site is not conserved in MmSVCT1 (Supplementary Fig. 8), and no strong lipid-like density near the UapA lipid binding site was found in MmSVCT1. The core domain contains a vitamin C binding site located between the half helices of TM3 and TM10 (Fig. 1c, e). Above the core domain, there is an elongated extracellular loop (EL2) between TM3 and TM4 (Fig. 1d, e), on which Cys136 forms a disulfide bond with Cys78 on EL1 between TM1 and TM2, contributing to the structural integrity. Both the apo and substrate-bound structures adopt an inward-open conformation, and a solvent-accessible channel sandwiched between the core domain and gate domain can reach the substrate from the cytoplasmic side (Fig. 1f and Supplementary Fig. 5). As in UapA, TM13 of MmSVCT1 contributes to the formation of the inward-facing translocation channel of the neighboring subunit (Supplementary Fig. 9), which may play a role in substrate release^30^.

### Extracellular interfaces

**Fig. 2:**
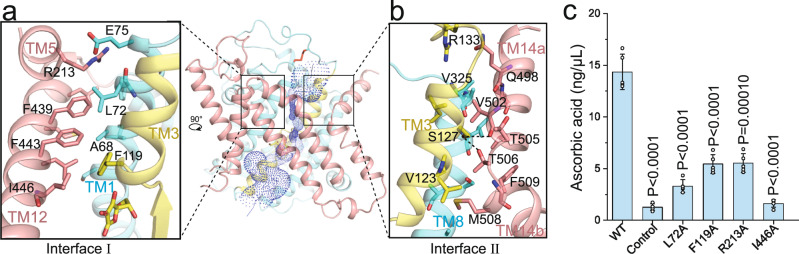
Extracellular interfaces of MmSVCT1. **a** One subunit of MmSVCT1 viewed from the gate domain with a zoomed-in view of interface I. The TM13 helix was removed for clarity. The central pore calculated by the program Hole is shown as a dotted mesh. **b** Magnified view of interface II. **c** Transport activity of MmSVCT1 variants with point mutations at the extracellular interface. Data points are the mean ± SEM (*n* = 5 independent experiments). *P* values from the two-tailed *t*-test are indicated on the bar chart. Source data are provided as a Source Data file.

Two tightly packed extracellular interfaces between the core domain and gate domain (interface I and II) comprise the closed extracellular gate of the inward-open structures of MmSVCT1 (Fig. 2a, b). The two interfaces are separated by a central pore with a small radius impermeable to the solvent (Fig. 2a and Supplementary Fig. 10). Localized above the substrate binding site, Interface I is predominantly formed by hydrophobic residues, including Phe119 from TM3 and multiple residues from TM1 and TM12. In addition, Arg213 from TM5 establishes polar interactions with the side chain of Glu75 and the carbonyl group of Phe71 from TM1 on the extracellular side (Fig. 2a), further stabilizing the interface. The alanine substitution of Leu72, Phe119, Arg213 or Ile446 on interface I substantially reduced the transport activity of MmSVCT1 (Fig. 2c), while their overall expression and plasma localization showed no major difference from WT MmSVCT1 (Supplementary Fig. 11). Consistent with our structural and functional analysis of interface I, mutations in TM5 and TM12 of UapA have been previously shown to affect the transport dynamics, substrate affinity, and specificity^33^. Interface II is formed by residues from TM3, TM8, TM14a, and TM14b (Fig. 2b), in which the side-chain hydroxyl group of Ser127 establishes two hydrogen bonds with the carbonyl group of Val502 and the side-chain hydroxyl group of Thr506. Interface II is important in that several residues on TM3 and TM8 directly participate in the formation of substrate and ion binding sites, as discussed below. The extensive interactions of the extracellular interfaces between the core domain and gate domain maintain MmSVCT1 structures in the inward-facing conformation.

### Vitamin C and sodium binding site

**Fig. 3:**
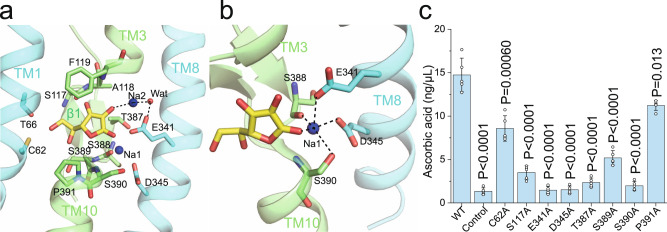
Vitamin C and sodium ion binding in MmSVCT1. **a** Residues in the proximity of the vitamin C molecule. Sodium ions are shown as blue spheres, and a potential water molecule (Wat) is shown as a red sphere. Interactions are indicated by dashed lines. **b** Coordinating residues of Na1. **c** Uptake activity of MmSVCT1 variants with point mutations at vitamin C and sodium ion binding sites. Data points are the mean ± SEM (*n* = 5 independent experiments). *P* values from the two-tailed *t*-test are indicated on the bar chart. Source data are provided as a Source Data file.

In the cryo-EM density maps of the substrate-bound structure, prominent nonprotein density can be observed in a pocket surrounded by TM1, TM3, TM8, and TM10 (Fig. 3a and Supplementary Fig. 12a, b). A vitamin C molecule can be reasonably placed into the density (Supplementary Fig. 12a, b), although an alternative conformation cannot be completely ruled out with the current local resolution. Previous in silico docking analysis has suggested similar substrate binding in human SVCT2^34^. Interestingly, some extra density can be observed outside the binding pocket between the core and gate domains, which is likely to be detergents or lipids (Supplementary Fig. 13), as seen in the UapA structure^30^. Most of the residues lining the binding site are polar residues and are highly conserved among SVCTs (Fig. 3a and Supplementary Fig. 1). TM3 and TM10 form a crossing above the substrate. As a result, Ser117 and Phe119 on the connecting loop between the β1 strand and TM3 helix directly interact with the substrate (Fig. 3a), while Ala118 forms hydrogen bonds with the side chain of Asn384 on TM10 (Supplementary Fig. 14a), stabilizing the loop. Below the substrate, residues in the sequence ^387^Thr-Ser-Ser-Ser, which immediately precede the NAT motif comprising the TM10 helix, establish multiple interactions with vitamin C (Fig. 3a), contributing to substrate specificity determination. Following the ^387^Thr-Ser-Ser-Ser sequence, Pro391 in MmSVCT1, the initial residue in the NAT motif, interacts with the vitamin C molecule with its bulky side chain through van der Waals contact (Fig. 3a). Consistent with our observation, previous studies have suggested that residues corresponding to Ser389 and Pro391 are key elements that distinguish vitamin C transporters from other nucleobase transporters^35,36^. In addition, Cys62 and Thr66 on TM1 interact with the substrate from one lateral side, and Glu341 and Asp345 on TM8 stabilize the sodium ions and substrate from the other side (Fig. 3a, b). We performed mutagenesis studies to evaluate the functional significance of these residues around the vitamin C binding site. While the overall expression and plasma localization showed no major difference with WT MmSVCT1 (Supplementary Fig. 11), all alanine substitutions (C62A, S117A, T387A, S389A, or P391A) profoundly reduced vitamin C uptake (Fig. 3c), indicating their critical roles in substrate recognition. The *K*_*m*_ of WT MmSVCT1 is close to that of human and rat SVCTs reported previously^15,18,20,21^ (Supplementary Fig. 15). The C62A and P391A mutants exhibited *K*_*m*_ and *V*_max_ values similar to that of WT MmSVCT1, while other mutants showed nearly no activity (Supplementary Fig. 15). Notably, the strong vitamin C density is absent in the apo map despite some unknown densities (Supplementary Fig. 12b, c). Moreover, there is no major structural change in the binding pocket upon substrate binding (Supplementary Fig. 14b), probably because the abundant hydrogen bonds in the unwound regions of TM3 and TM10 stabilize the pocket (Supplementary Fig. 14a). The vitamin C recognition of MmSVCT1 is different from that of the bacterial phosphorylation-coupled vitamin C transporter UlaA^37^.

The calculated surface electrostatic potential of the binding pocket in the MmSVCT1 protein is rather negative (Fig. 1f). Sodium ion binding, together with the positively charged ends of TM3 and TM10 dipoles, is thus required for the recognition of the negatively charged vitamin C (pKa = 4.70) at physiological pH (Fig. 3a). Two potential Na^+^ binding sites, designated as Na1 and Na2, were identified around the substrate binding pocket (Fig. 3a). The coordination of Na1 is provided by the main-chain carbonyl oxygen of Ser388 and side-chain oxygens of Glu341, Asp345, Ser390, and vitamin C (Fig. 3b), with coordination distances of 2.2–2.8 Å. Asp345 in MmSVCT1, corresponding to His245 in proton symporter UraA, was suggested to play a key role in sodium symport^28^, which is consistent with its direct Na^+^ coordination. Importantly, all mutations of the coordinating residues—E341A, D345A, and S390A—abolished transport activity (Fig. 3c), showing their functional significance. The ion at the tentative Na2 site is less well coordinated and interacts with the substrate and a potential water molecule that is stabilized by Glu341 (Fig. 3a).

### Transport mechanism

**Fig. 4:**
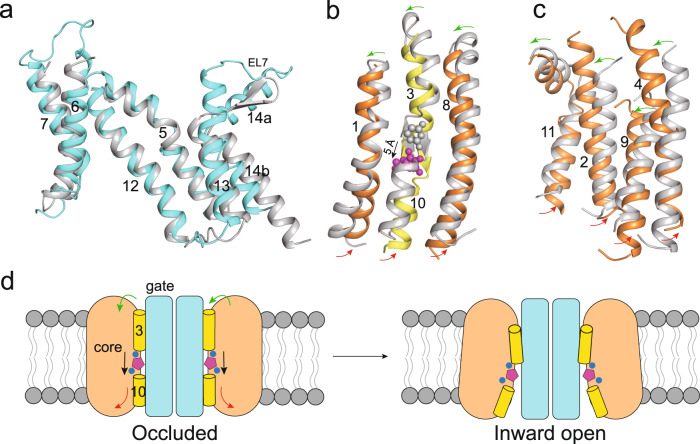
Transport mechanism of MmSVCT1. **a** Structural comparison of the gate domain between inward-open MmSVCT1 (cyan) and occluded UraA (gray). **b**, **c** Movements of the core domain of the inward-open MmSVCT1 (orange or yellow) and occluded UraA (gray) viewed from the gate domain. Vitamin C and uracil are shown as purple and gray spheres, respectively. The directions of movements of the substrate (black arrows), the extracellular segments (green arrows) and intracellular segments (red arrows) of the core domain helices are indicated. **d** Working model for the vitamin C transport of MmSVCT1. The purple pentagons and blue spheres indicate the substrate and sodium ions, respectively.

To understand the transport mechanism of MmSVCT1, we compared our inward-open MmSVCT1 structure with a previously reported occluded UraA structure^29^. The gate domain of MmSVCT1 and UraA can be aligned exceptionally well with a backbone RMSD of ~1.3 Å (Fig. 4a), rationalizing our analysis based on the comparison of two structures. The largest structural difference between the gate domains of the two structures is from the loop EL7 between TM13 and TM14 (Fig. 4a); two beta strands in the loop of UraA are replaced by elongated TM13 helix and TM14a in MmSVCT1. When superposed by the gate domains, the intracellular segments of the core domain helices undergo outwards movement from the occluded structure to the inward-open structure, resulting in the opening of the intracellular side of the MmSVCT1 structure (Fig. 4b–d). Along with that, the substrate bound in the core domain shows a downward shift of ~5 Å (Fig. 4b), which is consistent with an elevator mechanism first proposed for Glt_Ph_^38^. However, instead of a rigid-body movement, the core domain is also subjected to local structural arrangements: the extracellular segments of the core domain helices of MmSVCT1 undergo an anticlockwise rotation compared to their UraA counterparts (Fig. 4b, c). Thus, MmSVCT1 explores a mechanism combining an elevator-like shift with local structural changes to deliver the substrate (Fig. 4d).

## Discussion

Previous electrophysiological studies suggested an SVCT binding sequence of Na^+^, L-ascorbic acid, Na^+^^17,18^. We noticed that the substrate binding site is located at a position halfway across the membrane in the core domain, which is surrounded by multiple polar residues and a largely negative electrostatic surface. A sodium ion may first bind near the Na1 site, the stronger ion binding site, partly neutralizing Asp345 and/or Glu341 on TM8, which makes the substrate binding site more suitable for negatively charged vitamin C. Vitamin C binding is then followed by Na^+^ at the Na2 binding site, which is primarily coordinated by vitamin C. In line with our analysis, a similar transport mechanism of UapA has been proposed previously^39^, in which the symported proton neutralized the negative charge of D360 and E356 (corresponding to D341 and E345 in MmSVCT1) and then allowed substrate binding and transport. In the inward-facing conformation of MmSVCT1, the bound sodium ions are likely in a prerelease conformation, and their dissipation into the low-sodium cytosol would promote vitamin C release. Thus, the structural analyses of MmSVCT1 presented here rationalize previously reported electrophysiological data and provide critical insights into vitamin C and ion recognition as well as their cotransport.

MmSVCT1 functions as a homodimer with its gate domain forming the dimeric interface. This is similar to the microbial NAT/SLC23 transporters UraA^29^ and UapA^30^, anion exchange 1 (SLC4A1)^40^ and plant borate efflux transporter Bor1^41^, but different from the epithelial anion transporter SLC26A9^42,43^ and plant sulfate transporter AtSULTR4;1^44^, which are dimerized primarily with cytoplasmic domains. Structural comparison between the inward-open MmSVCT1 and the occluded UraA suggests that SVCTs transport vitamin C through an elevator mechanism (Fig. 4b, d). Relative to the gate domain, the substrate-bound core domain undergoes rotation with some local rearrangements to transport the substrate. The gate domain is very similar between the inward-open MmSVCT1 and the occluded UraA (Fig. 4a), which is likely because both structures have closed extracellular gates. We suspect that when the transporter switches to an outward-open conformation, the extracellular interfaces (Interfaces I and II) would be destroyed, and the helices in the gate domain, especially for TM12 in interface I containing a hydrophobic surface (Fig. 2a), would rearrange to adapt to the environment exposed to solvent. This can then lead to structural changes on the dimeric interface side of the gate domain. As the same conformation was observed for both subunits in all known dimeric SLC4/23 structures^29,30,40,41^ and MmSVCT1 structures in the current study, concurrent rearrangements of the dimeric gate domains may provide a mechanism for coordinating the two neighboring subunits. Indeed, the dimeric interface has been proven to be essential for the substrate transport of UraA^29^ and UapA^30^. Future work needs to determine SVCT structures in outward-facing and occluded states to pinpoint the detailed conformational changes during a transport cycle for this family of vitamin C transporters. Together, our structural studies offer important insights into vitamin C recognition and lay a foundation for further mechanistic studies.

## Methods

### Protein expression, purification and nanodisc reconstitution

MmSVCT1 (SLC23A1, NCBI accession: NP_035527.3) was cloned into a pEZT-BM vector with a C-terminal strep tag and heterologously expressed in HEK293F cells (Thermo Fisher Scientific, R79007) using the BacMam system (Thermo Fisher Scientific). The primers used in this study are provided as a Source Data file. Baculovirus was generated from Sf9 cells (Thermo Fisher Scientific, 11496015) and used to infect HEK293F cells at a volume ratio of 1:40 (virus:cell). HEK293F cells were cultured in suspension in 293F medium (Sino Biological Inc.) at 37 °C and 5% CO_2_. Sodium butyrate (10 mM) was added to the culture medium to boost protein expression. After 48–72 h, the cells were harvested by centrifugation at 5000 g, resuspended in buffer A (50 mM Tris, pH 8.0, 150 mM NaCl), and homogenized by sonication on ice. A protease inhibitor cocktail (containing 2 μg/ml DNase I, 0.5 μg/ml pepstatin, 2 μg/ml leupeptin, 1 μg/ml aprotinin, and 0.1 mM PMSF) was added to the buffer throughout the purification process. MmSVCT1 was extracted with 1% (w/v) n-dodecyl-β-d-maltopyranoside (DDM, Anatrace) supplemented with 0.2% (w/v) cholesteryl hemisuccinate (Sigma-Aldrich) by gentle agitation for 2 h. After extraction, the supernatant was collected after 60-min centrifugation at 30,000 g and purified with Strep-Tactin XT 4Flow resin (IBA). During resin washing, the buffer was gradually changed to buffer B (50 mM Tris, pH 8.0, 150 mM NaCl, and 0.03% GDN). The protein was then eluted with buffer B supplemented with 50 mM D-biotin, concentrated to a final volume of 500 μl, and further purified by size exclusion chromatography on a Superose 6 10/300 GL column (GE Healthcare) preequilibrated with buffer B.

Nanodisc reconstitution was performed by following a published protocol^45^. Peak fractions after size exclusion chromatography were pooled and mixed with MSPE3D1 and lipids (POPC: POPG: POPE = 3:1:1) at a molar ratio of 1:4:20 (MmSVCT1: MSPE3D1: lipid). After detergent removal by Bio-Beads SM2 (Bio-Rad), the sample was loaded onto a Superose 6 10/300 GL column preequilibrated with buffer A. Peak fractions of reconstituted MmSVCT1 were collected for cryo-electron microscopy analysis.

### Cryo-EM sample preparation and EM data acquisition

For the apo MmSVCT1 sample, cryo-EM grids were prepared by applying 4 μl of MmSVCT1 in GDN (~4.0 mg/ml) to a glow-discharged Quantifoil R2/1 200-mesh copper holey carbon film (Quantifoil, Micro Tools GmbH, Germany). Grids were blotted for 3.0 s under 100% humidity at 4 °C before being plunged into liquid ethane using a Mark IV Vitrobot (Thermo Fisher). For the substrate-bound MmSVCT1 sample, MmSVCT1 in nanodiscs (~3.0 mg/ml) was incubated with 1 mM ascorbic acid for 30 min on ice before grid freezing.

Micrographs were acquired on a Titan Krios microscope (Thermo Fisher) operated at 300 kV with a K3 Summit direct electron detector (Gatan). Images were recorded with EPU software (Thermo Fisher) in counting mode with a pixel size of 0.82 Å. The defocus range was set from −1.5 to −2.7 μm. Each micrograph was dose-fractionated to 30 frames under a dose rate of ~18.7 e^-^/Å^2^/s, with a total exposure time of 3 s.

### Image processing

Data processing was performed using the software package cyroSPARC^46^. Micrographs were motion corrected with patch motion correction. The CTF parameters of micrographs were estimated using patch CTF estimation. For the apo MmSVCT1 dataset, using templates selected from the 2D classification of 151 manually picked particles, 2,464,213 particles were automatically picked and extracted from the full dataset of 2789 micrographs. After 2D classification, a small dataset containing 83,323 particles was selected for ab-initio reconstruction to generate the reference maps. Then, a total of 614,477 particles resulting from 2D classification were used for heterogeneous refinement. Four rounds of heterogeneous refinement were performed using two reference maps, one with good secondary structural features and one with an empty micelle. After each heterogeneous refinement, particles assigned to the good class were selected for the subsequent heterogeneous refinement. A final dataset of 213,199 particles was then subjected to non-uniform refinement with C2 symmetry imposed, yielding a reconstruction at a resolution of 3.5 Å.

The dataset for substrate-bound MmSVCT1 in nanodiscs was processed similarly to that of apo MmSVCT1. In brief, 2,370,225 particles were picked and extracted from 4708 micrographs. After 2D classification, a total of 444,100 particles were selected for heterogeneous refinement. Three rounds of heterogeneous refinement were performed with two reference maps generated by ab-initio reconstruction from a small dataset of 88,107 particles. A dataset of 224,682 particles was then subjected to non-uniform refinement with C2 symmetry imposed, yielding a reconstruction at a resolution of 3.2 Å. To improve the resolution, one round of local refinement was performed with a soft mask around the protein density (excluding the belt-like density from the nanodisc), yielding a 2.8 Å resolution cryo-EM map. The 3.2 Å map with better peripheral loop density was used to build the overall structure of substrate-bound MmSVCT1 in nanodiscs, and the 2.8 Å map from local refinement was used to help determine the substrate conformation.

### Model building, refinement, and validation

Model building was conducted in Coot^47^, and an MmSVCT1 structure (AF-Q9Z2J0-F1) from the AlphaFold Protein Structure Database^48^ was used as an initial reference. Real-space model refinement^49^ and validation^50^ were performed in Phenix. For the substrate-bound state, residues 37–239, 258–534, and 550–576 are included in the final structure model; residues 1–36, 240–257, 535–549, and 577–605 are disordered and not modeled. The sugar extended from Asn151 has visible but weak density, so it was not modeled. For the apo state, the final structure model includes residues 43–239, 258–534, and 550–576. The statistics of the model geometries were generated using MolProbity^51^. The transport pathway of MmSVCT1 was predicted by the program HOLE^52^. The surface electrostatic potential was calculated by the program APBS in PyMol. Figures were prepared using PyMol^53^ and ChimeraX^54^ software. Q-scores characterizing atom resolvability in the map were calculated as previously described^55^.

### Ascorbic acid uptake assay

MmSVCT1 transport activity was assayed by measuring ascorbic acid uptake in HEK293F cells. In this assay, the ascorbic acid concentration was determined by using the Ferric Reducing/Antioxidant and Ascorbic Acid kit (Sigma, MAK075). Wild-type MmSVCT1 and variants containing a C-terminal GFP followed by a Strep tag were cloned into a pEZT-BM vector and transiently expressed in HEK293F cells using linear polyethylenimine Max (Polysciences) according to the manufacturer’s protocol. Cells were cultured in suspension in 293F medium (Sino Biological Inc.) at 37 °C and 5% CO_2_. Twenty-four hours after transfection, the medium was replaced with fresh 293F medium supplemented with 10 mM sodium butyrate. After an additional 24 h of culture, the cells were incubated in fresh HEK293F medium supplemented with 0.1 mM ascorbic acid at 37 °C for 30 min. For the inhibition assay, 0.1 mM diclofenamic acid was added. Approximately 2 × 10^6^ cells were then collected and washed twice with ice-cold PBS and rapidly homogenized in lysis buffer from the kit. After removing insoluble material by centrifuging at 13,000 × g for 10 min at 4 °C, the sample was added to a 96-well plate and incubated with a reaction mix for 2–3 min at room temperature. During this process, Fe^3+^ was reduced to Fe^2+^ by ascorbic acid present in the sample, resulting in a colorimetric product. A blank control was set simultaneously for each sample, in which ascorbic oxidase was added to oxidize the ascorbic acid, and its reading was subtracted from the sample reading to obtain the corrected measurement. Meanwhile, a series of ascorbic acid standards with 0 (blank), 2, 4, 6, 8, and 10 nmol/well were made to set up a standard curve. The absorbance at 593 nm was measured for each sample, and the amount of ascorbic acid was determined from the standard curve. All samples were run in 5 repeats.

The *K*_*m*_ and *V*_max_ of WT and MmSVCT1 mutants were determined by fitting the data to the Michaelis–Menten equation in Origin software. The initial transport velocities were measured at 1 min over a substrate concentration range of 0–200 μM. All experiments were repeated three times.

### Western blot and immunofluorescence imaging

To monitor the expression level and subcellular localization, WT and MmSVCT1 mutants containing a C-terminal GFP followed by a Strep tag were transfected into HEK293T cells (ATCC, #CRL-3216). The cells were maintained in DMEM (Cytiva) with 10% fetal bovine serum (HyClone) and 100 units/ml penicillin plus 100 µg/ml streptomycin. For western blot, cells were harvested 48 h after transfection and solubilized in a buffer containing 50 mM Tris, pH 8.0, 150 mM NaCl, and 1% DDM for 1 h at 4 °C. After centrifugation at 20,000 × g for 30 min, equal amounts of samples were resolved on an SDS-polyacrylamide gel and transblotted onto a PVDF membrane. The membrane was then probed with 1:2000 diluted mouse anti-StrepII-tag monoclonal antibody (ABclonal, AE066, Clone No. AMC0521) and 1:5000 diluted goat HRP-conjugated anti-mouse IgG (BBI Life Sciences, D110087).

Immunofluorescence imaging was performed following the published protocol^56^. HEK293T cells were grown on coverslips and transfected with MmSVCT1 variants. After 24 h, the cells were fixed with 3.7% formaldehyde in PBS buffer. DNA was stained with 4′,6-diamidino-2-phenylindole (DAPI) from Sigma. Images were collected via the FITC channel (for the GFP signal) or DAPI channel on a DeltaVision deconvolution microscope (GE Healthcare). Data were captured and processed using DeltaVision softWoRx software V6.5.2 (Applied Precision).

### Reporting summary

Further information on research design is available in the Nature Portfolio Reporting Summary linked to this article.

## Supplementary information

Supplementary Information

Reporting Summary

## Data Availability

The data that support this study are available from the corresponding authors upon request. Cryo-EM maps have been deposited in the Electron Microscopy Data Bank (EMDB) under accession codes EMD-34094 (Vitamin C-bound MmSVCT1) and EMD-34095 (apo MmSVCT1). Atomic coordinates have been deposited in the Protein Data Bank (PDB) under accession codes 7YTW (Vitamin C-bound MmSVCT1) and 7YTY (apo mMSVCT1). The source data underlying Figs. 1a, 2c and 3c and Supplementary Figs. 11a and 15 are provided as a Source Data file. The sequence of mouse SVCT1 is available through NCBI accession NP_035527.3. Source Data are provided with this paper.

## Source data

Source Data
