# Supplementary material for: Structural basis of vitamin C recognition and transport by mammalian SVCT1 transporter
Source: Nat Commun. 2023 Mar 13;14:1361. doi: 10.1038/s41467-023-37037-3 (PMC10011568; doi:10.1038/s41467-023-37037-3)
Supplement: Supplementary file 1 — Supplementary Information [file 41467_2023_37037_MOESM1_ESM.pdf]

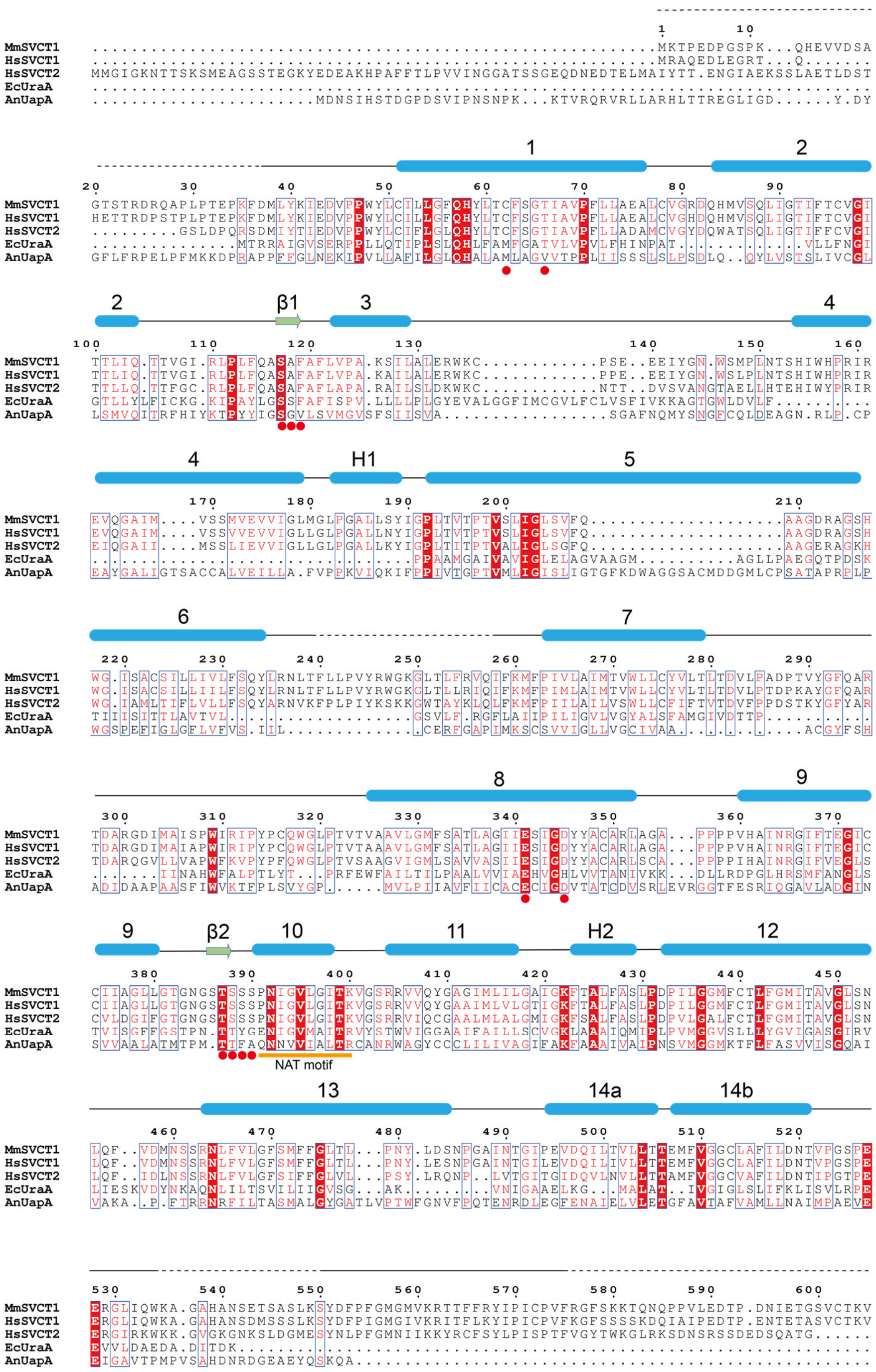

Supplementary Figure 1 | Sequence alignment of MmSVCT1, human SVCTs (HsSVCT1-2), *E. coli* UraA (EcUraA), and *Aspergillus nidulans* UapA (AnUapA). Secondary structure assignments are based on the substrate-bound mouse SVCT1 structure. Red dots mark the residues surrounding the substrate. The NAT motif is indicated.

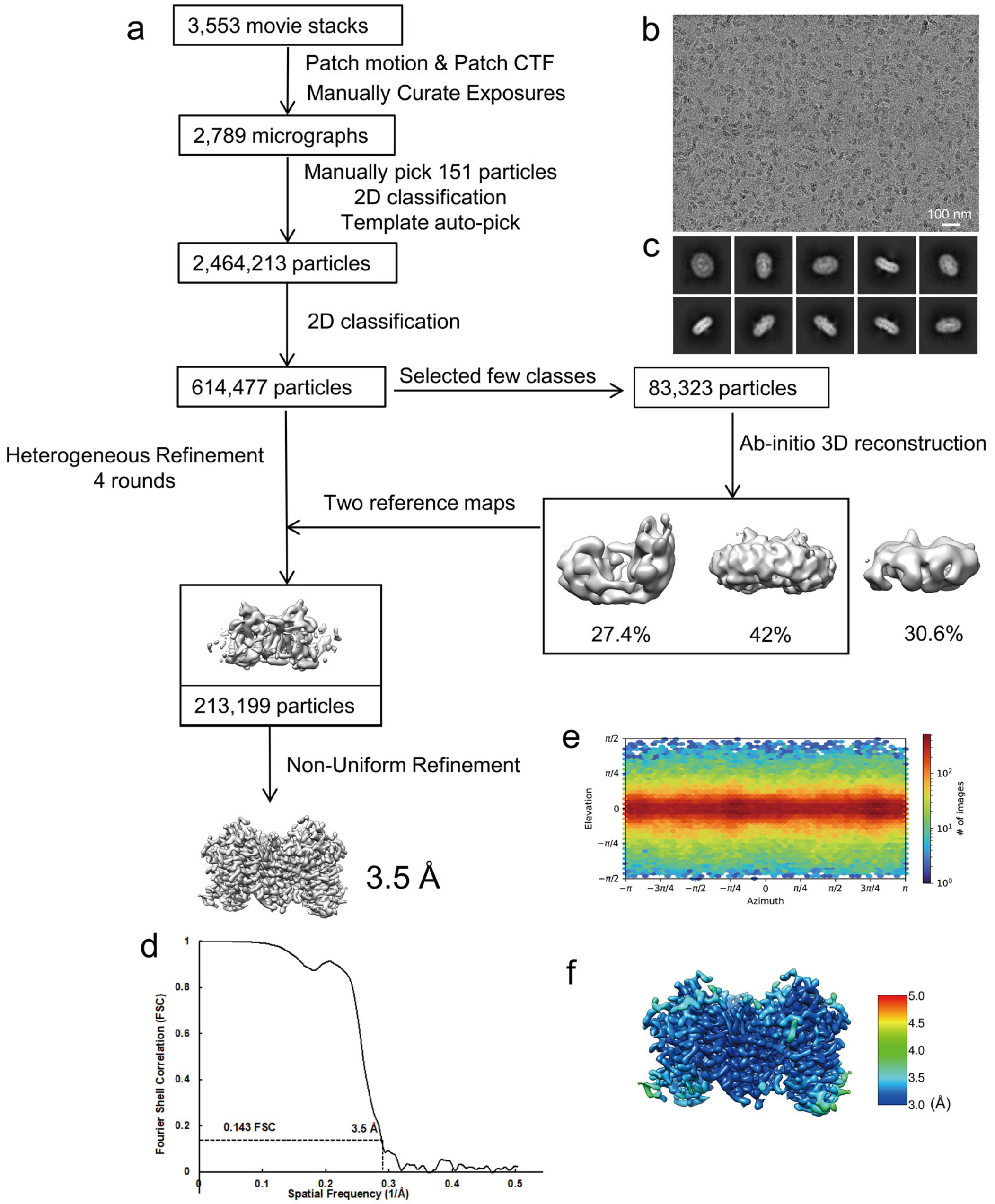

Supplementary Figure 2 | Single-particle cryo-EM data processing of apo MmSVCT1. a, Workflow of the data processing. **b,** Representative electron micrograph of apo MmSVCT1. **c,** Reference-free 2D class averages of computationally extracted particles. **d,** Gold standard FSC plot for the final 3D reconstruction. **e,** Euler angle distribution of the particle images. **f,** Resolution map for the 3D reconstruction calculated in cryoSPARC.

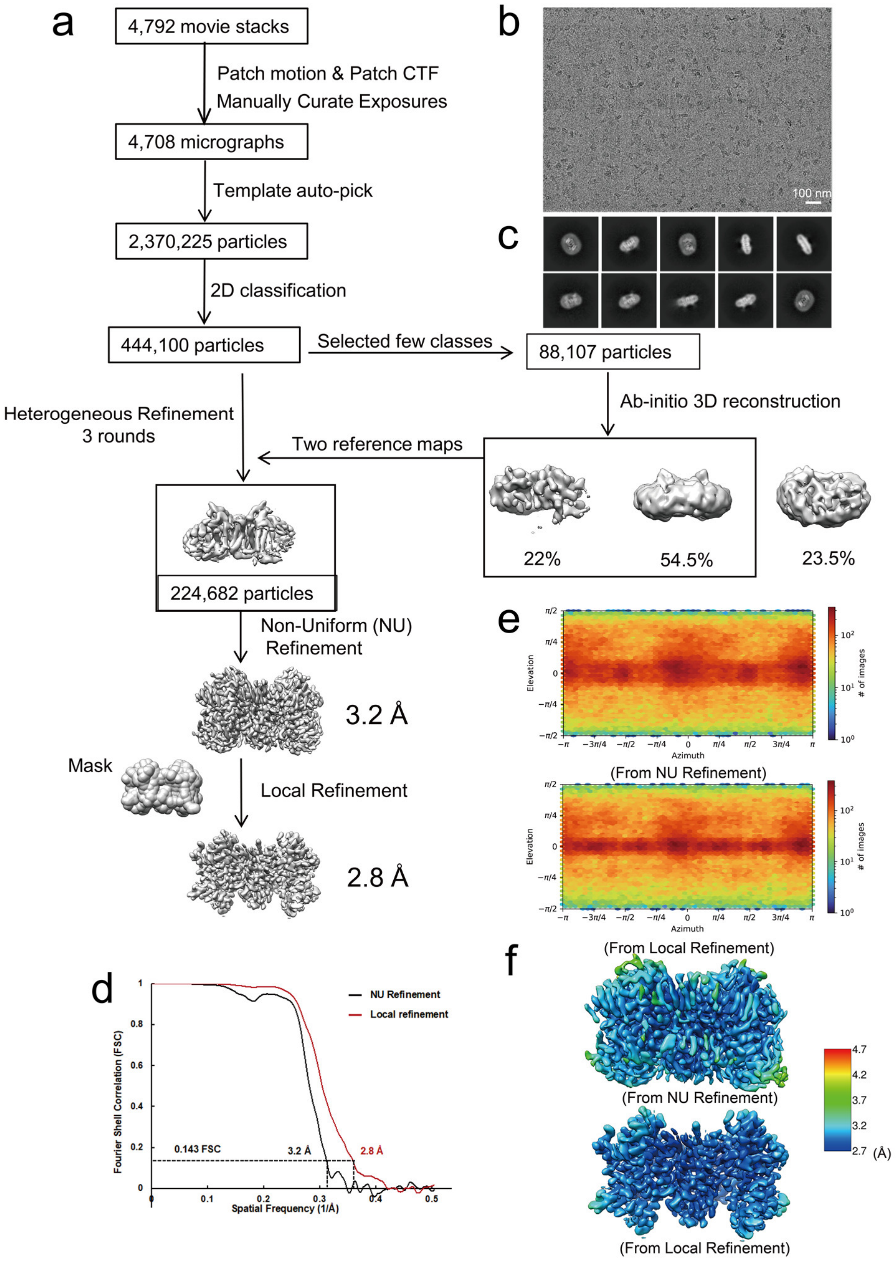

Supplementary Figure 3 | Single-particle cryo-EM data processing of substrate-bound MmSVCT1. **a,** Workflow of the data processing. **b,** Representative electron micrograph of substrate-bound MmSVCT1. **c,** Reference-free 2D class averages of computationally extracted particles. **d,** Gold standard FSC plots for the final 3D reconstruction. **e,** Euler angle distribution of the particle images for the 3D reconstructions from NU refinement (upper) and local refinement

(lower). **f,** Resolution map for the 3D reconstructions from NU refinement (upper) and local refinement (lower).

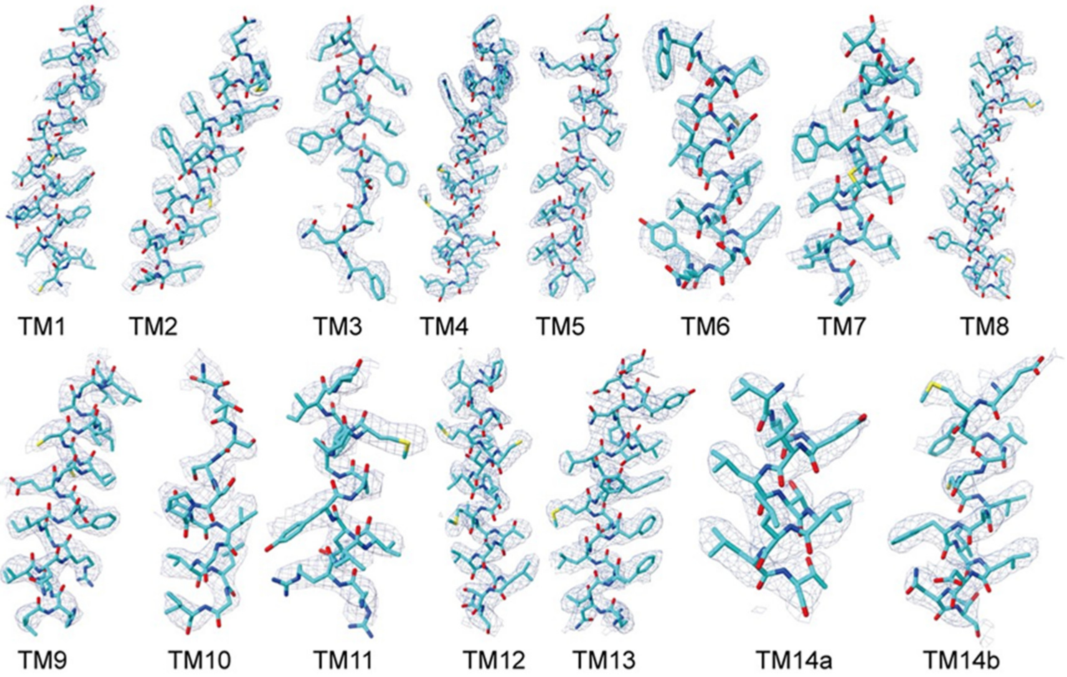

Supplementary Figure 4 | Sample electron microscopy density maps (grey mesh) for substrate-bound MmSVCT1. Refined coordinates are shown as sticks.

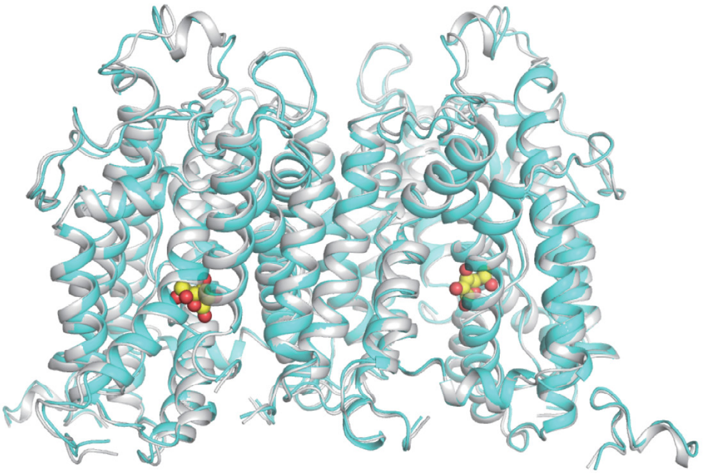

Supplementary Figure 5 | Structure of MmSVCT1. Superposition of the overall structures of apo MmSVCT1 (grey) and substrate-bound MmSVCT1 (cyan). Vitamin C is shown as spheres.

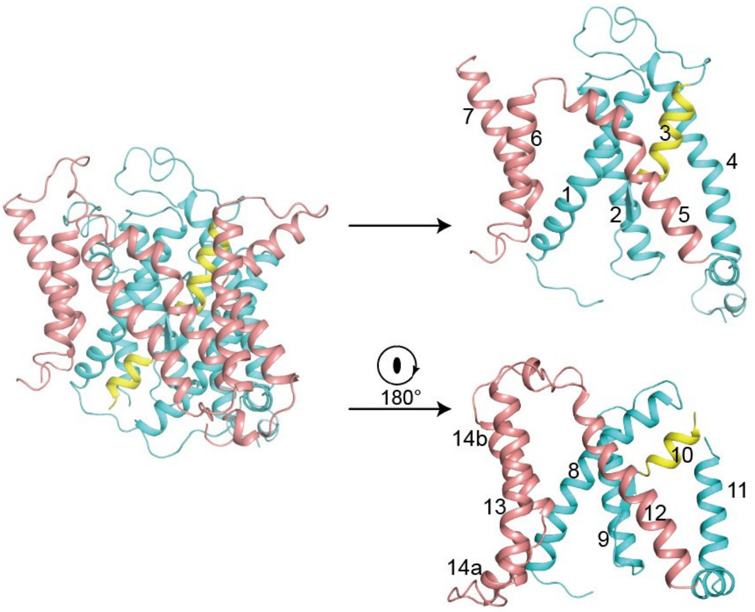

Supplementary Figure 6 | Structural repeats (TM1-7 and TM8-14) in MmSVCT1. TM8-14 (bottom right) is shown after an ~180° rotation around a pseudo two-fold axis parallel to the membrane.

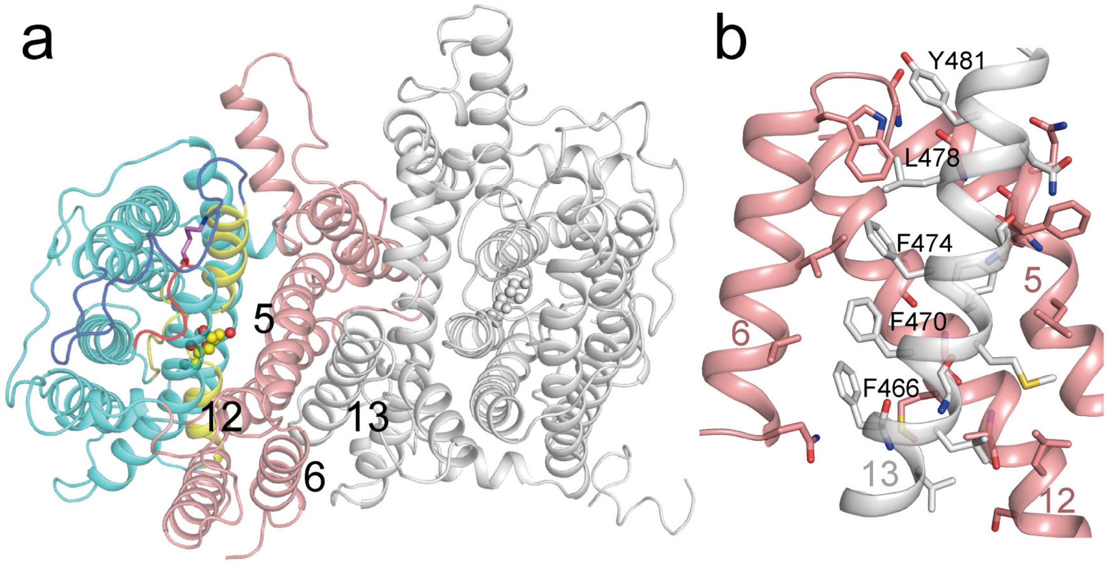

Supplementary Figure 7 | Dimeric interface of MmSVCT1. a, Dimeric MmSVCT1 viewed from the extracellular side. **b,** Residues involved in the dimeric interface are shown as sticks.

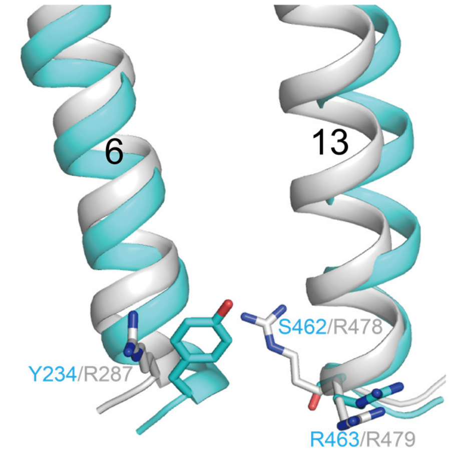

Supplementary Figure 8 | Structural alignment of MmSVCT1 (cyan) and UapA (grey) around the lipid binding site of UapA. Arg287, Arg478 and Arg479 form a lipid binding site in UapA.

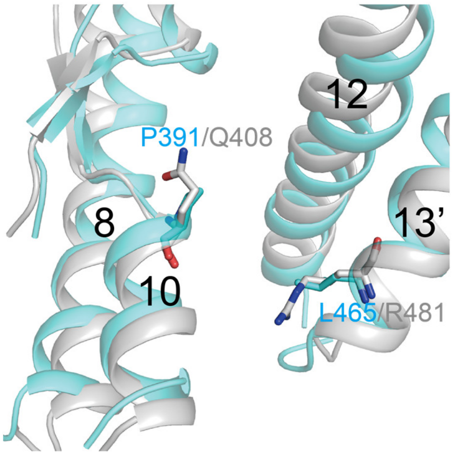

Supplementary Figure 9 | Structural alignment of the substrate release channel of MmSVCT1 (cyan) and UapA (grey). TM13’ is from the opposing subunit.

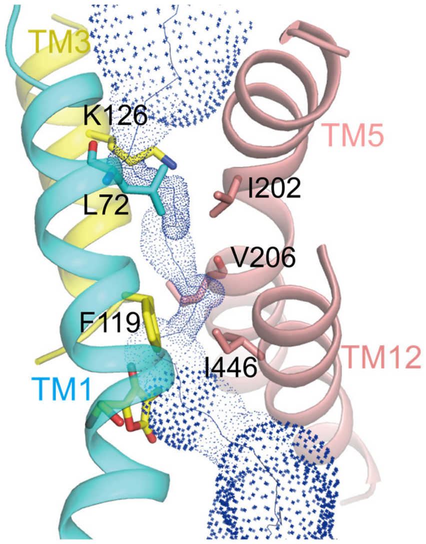

Supplementary Figure 10 | Central pore of MmSVCT1. The central pore is shown as a dotted mesh. Residues forming the narrowest constriction sites are shown as sticks.

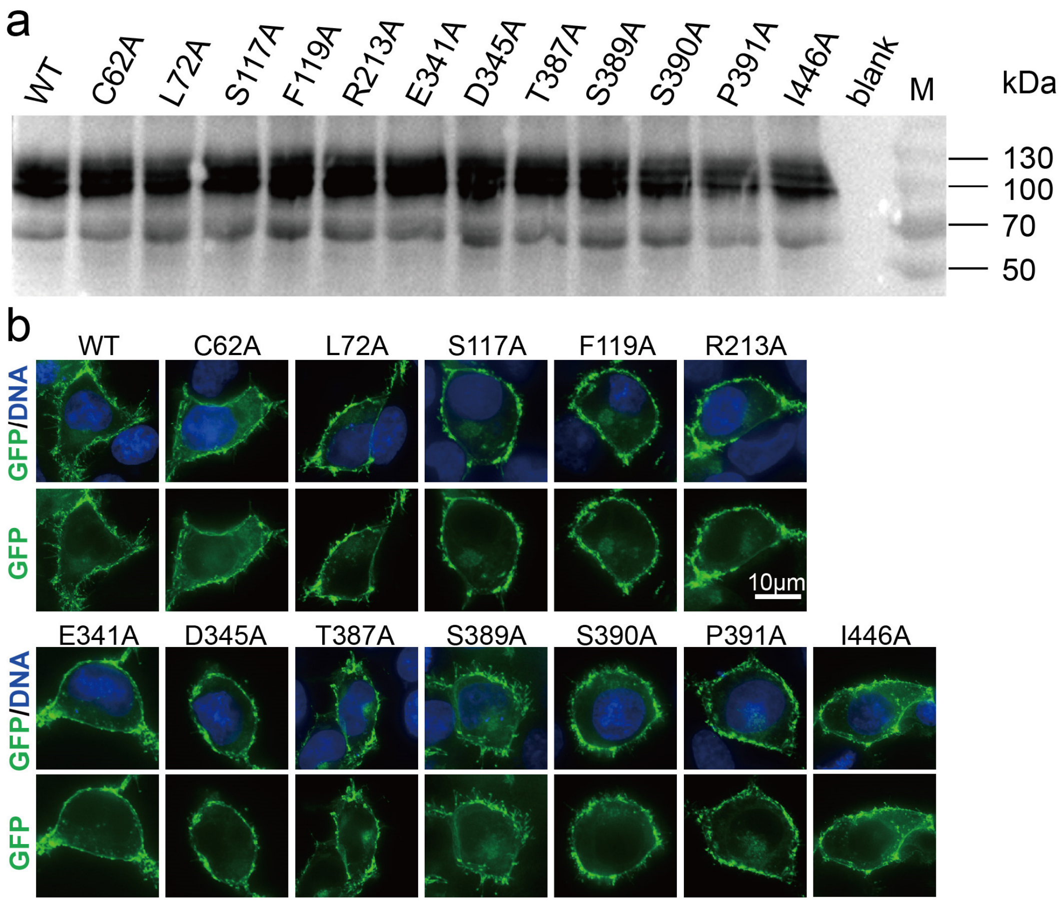

Supplementary Figure 11 | The expression and localization of WT and MmSVCT1 mutants. a, The expression levels of MmSVCT1 variants examined by western blot. Source data are provided as a Source Data file. **b,** Representative immunofluorescence images of HEK293T cells expressing GFP-tagged WT and MmSVCT1 mutants. DNA was stained with DAPI. The experiments were repeated three times independently with similar results.

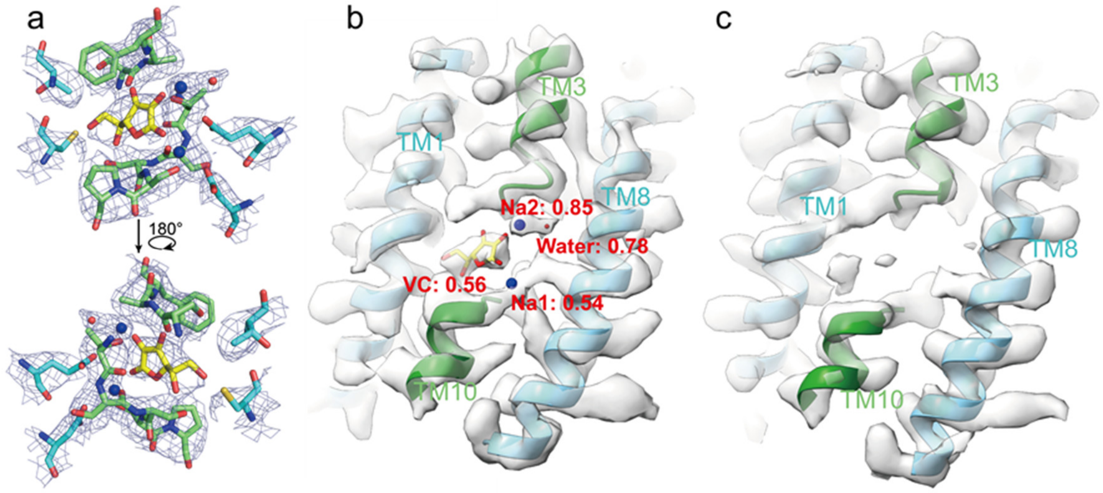

Supplementary Figure 12 | Electron microscopy density maps for the substrate binding site. **a,** Two views of density maps of the substrate binding site generated by PyMol from the substrate-bound map. Vitamin C (yellow sticks), sodium ions (blue spheres), and water (a red sphere) are shown. **b, c,** Density maps of the substrate binding site from the substrate-bound map (**b**) and apo map (**c**) generated by ChimeraX. Vitamin C (VC), sodium ions and water are displayed with Q-scores indicated.

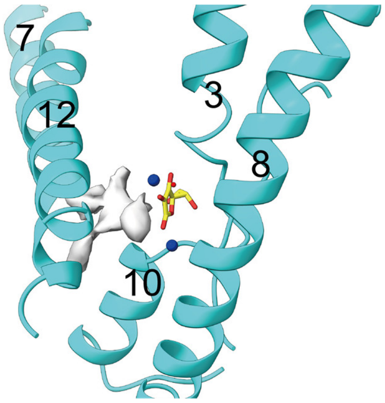

Supplementary Figure 13 | The nonprotein densities (grey) outside the binding pocket between the core and gate domains.

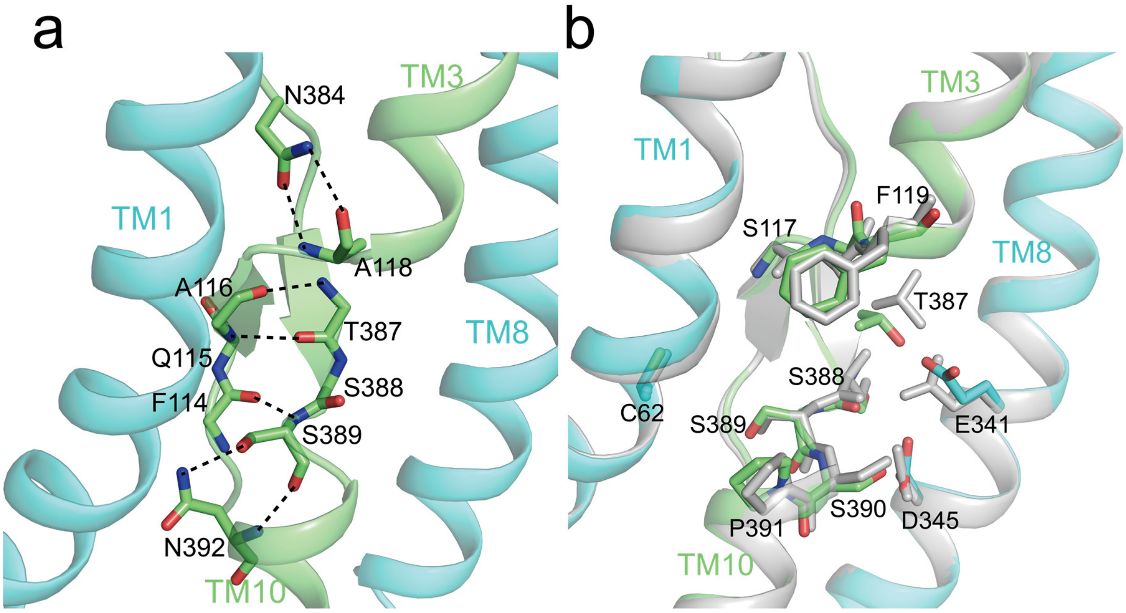

Supplementary Figure 14 | Vitamin C binding site. a, Hydrogen bond network (dashed lines) in the unwound regions of TM3 and TM10. The side chains of some residues are omitted for clarity. **b,** Structural comparison at the vitamin C binding site between the substrate-bound (cyan or green) and apo (grey) states.

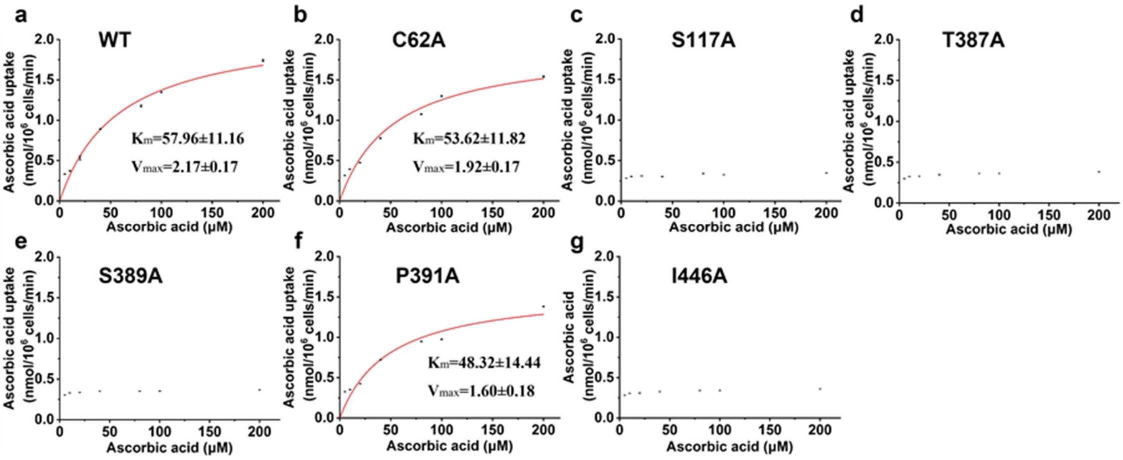

Supplementary Figure 15 | The initial rate of ascorbic acid uptake versus concentrations of WT and MmSVCT1 mutants. The data were fitted by the Michaelis–Menten equation. $K_{m,}$ the Michaelis constant in μM; $V_{max,}$ the maximum uptake rate in nmol/10^6 cells/min. The data for the S117A, T387A, S389A, and

I446A mutants were not fitted due to the weak signals. Data points are the mean± s.e.m. (n=3 independent experiments). Source data are provided as a Source Data file.

Supplementary information Table 1

Cryo-EM data acquisition, reconstruction and model refinement statistics.

	Vitamin C bound MmSVCT1 (EMDB-EMD-34094) (PDB-7YTW)	Apo MmSVCT1 (EMDB-EMD-34095) (PDB-7YTY)
**Data collection and processing**		
Microscope/Detector	K3	K3
Voltage (kV)	300	300
Electron exposure ($e^-/Å^2$)	55	55
Defocus range (μm)	-1.5 to -2.7	-1.5 to -2.7
Pixel size (Å)	0.82	0.82
Symmetry imposed	*C*2	*C*2
Initial particle images	2,370,225	2,464,213
Final particle images	224,682	213,199
Map resolution (Å)	3.2	3.5
FSC threshold	0.143	0.143
**Refinement**		
Initial model used	Alphafold: AF-Q9Z2J0-F1	Alphafold: AF-Q9Z2J0-F1
Model resolution (Å)	3.3	3.7
FSC threshold	0.5	0.5
Model composition		
Non-Hydrogen atoms	7,744	7,608
Protein residues	1014	1002
B factors ($Å^2$)		
Protein	47.37	56.27
Ligand	31.62	---
R.m.s. deviations		
Bond lengths (Å)	0.008	0.009
Bond angles (°)	1.112	1.172
Validation		
MolProbity score	1.80	1.69
Clashscore	8.32	7.48
Poor Rotamers (%)	0.00	0.00
Ramachandran plot		
Favored (%)	95.01	95.96
Allowed (%)	4.99	4.04
Disallowed (%)	0.00	0.00
