# Supplementary material for: Structural basis of vitamin C recognition and transport by mammalian SVCT1 transporter
Source: Nat Commun. 2023 Mar 13;14:1361. doi: 10.1038/s41467-023-37037-3 (PMC10011568; doi:10.1038/s41467-023-37037-3)
Supplement: Supplementary file 2 — Reporting Summary [file 41467_2023_37037_MOESM2_ESM.pdf]

nature portfolio

Corresponding author(s): Ji She, Kaiming Zhang

Last updated by author(s): Feb 6, 2023

# Reporting Summary

Nature Portfolio wishes to improve the reproducibility of the work that we publish. This form provides structure for consistency and transparency in reporting. For further information on Nature Portfolio policies, see our Editorial Policies and the Editorial Policy Checklist.

## Statistics

For all statistical analyses, confirm that the following items are present in the figure legend, table legend, main text, or Methods section.

| n/a | Confirmed | |
|---|---|---|
| ☐ | ☒ | The exact sample size (*n*) for each experimental group/condition, given as a discrete number and unit of measurement |
| ☐ | ☒ | A statement on whether measurements were taken from distinct samples or whether the same sample was measured repeatedly |
| ☐ | ☒ | The statistical test(s) used AND whether they are one- or two-sided<br>*Only common tests should be described solely by name; describe more complex techniques in the Methods section.* |
| ☒ | ☐ | A description of all covariates tested |
| ☒ | ☐ | A description of any assumptions or corrections, such as tests of normality and adjustment for multiple comparisons |
| ☐ | ☒ | A full description of the statistical parameters including central tendency (e.g. means) or other basic estimates (e.g. regression coefficient) AND variation (e.g. standard deviation) or associated estimates of uncertainty (e.g. confidence intervals) |
| ☐ | ☒ | For null hypothesis testing, the test statistic (e.g. *F*, *t*, *r*) with confidence intervals, effect sizes, degrees of freedom and *P* value noted<br>*Give P values as exact values whenever suitable.* |
| ☒ | ☐ | For Bayesian analysis, information on the choice of priors and Markov chain Monte Carlo settings |
| ☒ | ☐ | For hierarchical and complex designs, identification of the appropriate level for tests and full reporting of outcomes |
| ☒ | ☐ | Estimates of effect sizes (e.g. Cohen's *d*, Pearson's *r*), indicating how they were calculated |

*Our web collection on statistics for biologists contains articles on many of the points above.*

## Software and code

Policy information about availability of computer code

| | |
|---|---|
| Data collection | EPU(2.12), softWoRx(v6.5.2) |
| Data analysis | cryoSPARC (v3.3.2); Coot(v0.9.4.1); AlphaFold(); UCSF ChimeraX(v1.2); Phenix(v1.18.2);Pymol(v2.5.0);MolProbity(part of Phenix v1.18.2);HOLE (v2beta.002);APBS(part of Pymol2.4.0) |

For manuscripts utilizing custom algorithms or software that are central to the research but not yet described in published literature, software must be made available to editors and reviewers. We strongly encourage code deposition in a community repository (e.g. GitHub). See the Nature Portfolio guidelines for submitting code & software for further information.

## Data

Policy information about availability of data

All manuscripts must include a data availability statement. This statement should provide the following information, where applicable:

- Accession codes, unique identifiers, or web links for publicly available datasets
- A description of any restrictions on data availability
- For clinical datasets or third party data, please ensure that the statement adheres to our policy

The sequence of mouse SVCT1 is available through NCBI accession NP_035527.3. Cryo-EM maps of mouse SVCT1 in this study with its associated atomic models have been deposited in the wwPDB OneDep System under EMD accession codes EMD-34094 [https://www.ebi.ac.uk/pdbe/entry/emdb/EMD-34094] and EMD-34095 [https://www.ebi.ac.uk/pdbe/entry/emdb/EMD-34095] and PDB ID codes 7YTW [http://doi.org/10.2210/pdb7YTW/pdb] and 7YTY [http://doi.org/10.2210/pdb7VTY/pdb]. The source data underlying Figures 1a, 2c, 3c, Supplementary Figure 11a, and Supplementary Figure 15 are provided as a Source

Data file.

## Human research participants

Policy information about studies involving human research participants and Sex and Gender in Research.

| | |
|---|---|
| Reporting on sex and gender | N/A |
| Population characteristics | N/A |
| Recruitment | N/A |
| Ethics oversight | N/A |

Note that full information on the approval of the study protocol must also be provided in the manuscript.

# Field-specific reporting

Please select the one below that is the best fit for your research. If you are not sure, read the appropriate sections before making your selection.

☒ Life sciences ☐ Behavioural & social sciences ☐ Ecological, evolutionary & environmental sciences

For a reference copy of the document with all sections, see nature.com/documents/nr-reporting-summary-flat.pdf

# Life sciences study design

All studies must disclose on these points even when the disclosure is negative.

| | |
|---|---|
| Sample size | No statistical methods were used to predetermine sample size. For the transport assays, experiments were performed at least three independent times. The sample size was determined based on the reproducibility of the current recording. The data size of cryoEM was determined by he availability of the microscope time and the particle density on the grids. |
| Data exclusions | During cryoEM data processing, only the particles that give rise to homogeneous density were selected and used in the final reconstruction. |
| Replication | All experimental findings were reliably reproduced. All experiments were repeated at least three times. |
| Randomization | For structure refinement, all particles were randomly split into two groups. For the transport assays, randomization is not relevant as no group allocation were performed. |
| Blinding | Investigators were not blinded to group allocation, because no grouping was needed. |

# Reporting for specific materials, systems and methods

We require information from authors about some types of materials, experimental systems and methods used in many studies. Here, indicate whether each material, system or method listed is relevant to your study. If you are not sure if a list item applies to your research, read the appropriate section before selecting a response.

## Materials & experimental systems

| n/a | Involved in the study | |
|---|---|---|
| ☐ | ☒ | Antibodies |
| ☐ | ☒ | Eukaryotic cell lines |
| ☒ | ☐ | Palaeontology and archaeology |
| ☒ | ☐ | Animals and other organisms |
| ☒ | ☐ | Clinical data |
| ☒ | ☐ | Dual use research of concern |

## Methods

| n/a | Involved in the study | |
|---|---|---|
| ☒ | ☐ | ChIP-seq |
| ☒ | ☐ | Flow cytometry |
| ☒ | ☐ | MRI-based neuroimaging |

## Antibodies

| | |
|---|---|
| Antibodies used | mouse anti StrepII-tag monoclonal antibody (ABclonal, AE066, CloneNo. AMC0521) and goat HRP-conjugated goat anti-mouse IgG (BBI Life Sciences, D110087-0025) |
| Validation | The mouse anti StrepII-tag monoclonal antibody has been successfully used in western blot (https://abclonal.com/index.php?s=/ |

catalog-antibodies/MouseantiStrepIITagmAb/AE066). The goat HRP-conjugated goat anti-mouse IgG has been successfully used in western blot (https://www.sangon.com/productDetail?productInfo.code=D110087).

## Eukaryotic cell lines

Policy information about cell lines and Sex and Gender in Research

| | |
|---|---|
| Cell line source(s) | HEK293F (Thermo Fisher Scientific, R79007) ; Sf9 (Thermo Fisher Scientific, 11496015) ; HEK293T(ATCC, #CRL-3216). |
| Authentication | Cell line authentication was not performed. |
| Mycoplasma contamination | Cell lines were not tested for mycoplasma contamination. |
| Commonly misidentified lines (See ICLAC register) | No commonly misidentified cell lines were used. |
